# Low tidal volume protects pulmonary vasomotor function from “second-hit” injury in acute lung injury rats

**DOI:** 10.1186/1465-9921-13-77

**Published:** 2012-09-06

**Authors:** Chun Pan, Jianqiang Wang, Wei Liu, Ling Liu, Liang Jing, Yi Yang, Haibo Qiu

**Affiliations:** 1 Department of Critical Care Medicine, Zhongda Hospital, Southeast University Medical School, Nanjing, Jiangsu, China; 2 Department of Critical Care Medicine, Jintan Hospital, Changzhou, Jiangsu, China; 3 School of pharmacy, China Pharmaceutical University, Nanjing, Jiangsu, China; 4 Department of Anesthesiology, Zhongda Hospital, Southeast University, Nanjing, Jiangsu, China

**Keywords:** Endothelium, Mechanical ventilation, Vascular reactivity, Vascular injury, Lung injury, Pulmonary hypertension

## Abstract

**Background:**

Sepsis could induce indirect acute lung injury(ALI), and pulmonary vasomotor dysfunction. While low tidal volume is advocated for treatment of ALI patients. However, there is no evidence for low tidal volume that it could mitigate pulmonary vasomotor dysfunction in indirect ALI. Our study is to evaluate whether low tidal volume ventilation could protect the pulmonary vascular function in indirect lipopolysaccharide (LPS) induced acute lung injury rats.

**Methods:**

An indirect ALI rat model was induced by intravenous infusion of LPS. Thirty rats (n = 6 in each group) were randomly divided into (1)Control group; (2) ALI group; (3) LV group (tidal volume of 6mL/kg); (4) MV group (tidal volume of 12mL/kg); (5)VLV group (tidal volume of 3mL/kg). Mean arterial pressure and blood gas analysis were monitored every 2 hours throughout the experiment. Lung tissues and pulmonary artery rings were immediately harvested after the rats were bled to be killed to detect the contents of endothelin-1 (ET-1), endothelial nitric oxide synthase (eNOS) and TNF-α. Acetylcholine (Ache)-induced endothelium-dependent and sodium nitroprusside (SNP)-induced endothelium-independent relaxation of isolated pulmonary artery rings were measured by tensiometry.

**Results:**

There was no difference within groups concerning blood pressure, PaCO_2_ and SNP-induced endothelium-independent relaxation of pulmonary artery rings. Compared with MV group, LV group significantly reduced LPS-induced expression of ET-1 level (113.79 ± 7.33pg/mL vs. 152.52 ± 12.75pg/mL, *P* < 0.05) and TNF-α (3305.09 ± 334.29pg/mL vs.4144.07 ± 608.21pg/mL, *P* < 0.05), increased the expression of eNOS (IOD: 15032.05 ± 5925.07 vs. 11454.32 ± 6035.47, *P* < 0.05). While Ache (10^-7^mol/L-10^-4^mol/L)-induced vasodilatation was ameliorated 30% more in LV group than in MV group.

**Conclusions:**

Low tidal volume could protect the pulmonary vasodilative function during indirect ALI by decreasing vasoconstrictor factors, increasing expressions of vasodilator factors in pulmonary endothelial cells, and inhibiting inflammation injuries.

## Background

Acute lung injury (ALI) is a common clinical syndromes, and acute respiratory distress syndrome (ARDS) is a more severe ALI form. ALI/ARDS could induce pathophysiologic mechanisms of alveolar collapse, hyoxemia and vascular dysfunction [1,2]. Animal and clinical study found that ALI/ARDS could induce pulmonary vascular dysfunction, and pulmonary hypertension is independently associated with poor outcomes in ALI patients [2,3], and there was other study showed that sepsis could decrease pulmonary endothelium derived vasodilatation substances release, and injure pulmonary vasodilatation [4].

Mechanical ventilation is an important supportive strategy for patients with ALI/ARDS. While some animal studies demonstrated that high tidal volumes caused pulmonary edema, diffused alveolar damage, pulmonary hypertension and ventilator-induced lung injury (VILI) [5,6]. Nin and his colleagues infered that large tidal volume could decrease pulmonary artery relaxation of normal rats by inflammatory injuring pulmonary artery smooth muscles or decreasing vasorelaxing NO bioactivity [5]. Injurious ventilation is considered to be “second hit” for indirect ALI/ARDS patients. Clinical studies reported that low tidal volume (6mL/kg) could improve outcomes for ALI/ARDS patients with mechanical ventilation [7]. But, the mechanisms that account for the protective effects of low tidal volume on pulmonary vascular dysfunction from indirect ALI/ARDS patients are not fully understood.

Endotoxin, tissue stretch and fracture could be transduced into inflammatory signals to injure pulmonary endothelium. Endothelial cell injury is an important feature of ALI/ARDS, which can increase permeability of endothelial cells and cytokine release [8]. Imbalance of vasoconstrict and vasodilate cytokines derived from damaged endothelium could change pulmonary vasomotor tone [9,10].

The present study examined: (1) the mechanisms of sepsis induced the pulmonary vasodilation dysfunction, (2) the effects of tidal volume on lung vascular endothelial and pulmonary vasomotor tone in system inflammation. Most of the previous studies used either only endotoxin or mechanical ventilation in examining the effects of sepsis on endothelial functions in pulmonary arteries. In the present study, different tidal volumes based on sepsis which imitate the clinical condition of indirect ALI/ARDS were selected.

## Method

### Animal preparation and experimental protocol

This study was approved by the Ethics Committee of Southeast University Medical School, Nanjing, China. All animals received humane care in compliance with the “Principles of Laboratory Animal Care” formulated by the National Society for Medical Research and the “Guide for the Care and Use of Laboratory Animals” which was prepared by China National Academy of Sciences.

A total of 30 Sprague Dawley rats (240–320g) were randomly assigned into 5 main groups. Animals were anaesthetized with an intravenous injection of sodium pentobarbital (50 mg/kg) (Shanghai Chemical Reagent Co., China) via the femoral vein. Respiratory rate was adjusted to maintain arterial pH between 7.30 and 7.45. Systemic arterial pressure was monitored continuously and blood gas analysis was taken every 2 hours. Rats were received Halics lipopolysaccharide (LPS) (6mg/kg) (Escherichiacoli O111: B4, Sigma Chemical CO, St. Louis, MO) intravenously which suspended in saline solution with total volume equal to 0.5 mL for the lung injury model [11], and in the control (C) group, sterile saline solution (0.9% NaCl) was administrated at the same volume (0.5mL). After 1 hour, except for control group, ALI rats were divided randomly to four groups for an additional 5 hours: (1) ALI group, rats were placed supine and 100% oxygen was given by mask. (2) LV group, ALI rats were placed supine and ventilated through a tracheotomy tube (15-gauge luer stub adapter) with a volume-controlled ventilator (inspira ASV55-7058, Harvard Apparatus, USA) at the following settings: tidal volume (Vt) of 6mL/kg, positive end-expiratory pressure (PEEP) of 5cmH_2_O, 100% FiO_2_, respiratory rate at 40-60 breaths/min. (3) MV group, ALI rats were ventilated for 5 hours with large tidal volume (PEEP 5cmH_2_O, Vt 12mL/kg, FiO_2_ 100%, respiratory rate 40-60 breaths/min). (4) VLV group, ALI rats were ventilated for 5 hours with very low tidal volume (PEEP 5cmH_2_O, Vt 3mL/kg, FiO_2_ 100%, respiratory rate 40-60 breaths/min). 6 hours after injection of LPS and sterile saline, animals were sacrificed by exsanguinating. The lungs and pulmonary artery rings were immediately harvested for subsequent measurements.

### Histological and morphometric analysis

Fresh right lung lobus intermedius tissues were immediately fixed in 10% formalin. Slides were stained with hematoxylin and eosin, and then scored using a semiquantitative scoring system blinded to the treatment group. Edema, alveolar and interstitial inflammation, alveolar and interstitial hemorrhage, atelectasis, and hyaline membrane formation were each scored on a 0 to 4 point scale [12]: no injury = score of 0; injury in 25% of the field = score of 1; injury in 50% of the field = score of 2; injury in 75% of the field = score of 3; and injury throughout the field = score of 4. Ten microscopic fields from each slide were analyzed. The sums of tissue slides were averaged to evaluate the severity of lung injury.

### Endothelin-1 (ET-1) concentration and endothelial nitric oxide synthase (eNOS) expression

The concentration of ET-1 from supernatants derived from lung tissue homogenates was assessed by a radio-immunity kit (Center of radio-immunity of PLA General Hospital, Beijing, China) according to the manufacturer's instructions. The sensitivity of the assay was 5 pg/mL.

Expression of eNOS in lung tissues was assessed by immunohistochemistry. The antibodies used were monoclonal antibodies for eNOS (RB-1711-P1; Neomarkers, Fremont, CA, diluted 1:100). The lung slides were examined with an Olympus AX-70 microscope. Dark brown staining of the lung tissues indicates eNOS-positive cells. Image-Pro Plus 6.0 software was used to semi-quantitatively evaluate the expression of eNOS.

### Isolated vessel tension studies

At the end of the assigned protocol, the rats were killed by exsanguinating. The main pulmonary artery (PA) was immediately harvested and was carefully cleaned from adhering perivascular tissues, resulting in a ring of 1.5 to 2.0 mm length and 0.5 to 1.0 mm diameter. The vessel ring was suspended horizontally between two stirrups in organ chamber filled with 10mL of modified Krebs-Ringer bicarbonate solution (mmol/L: 118.3 NaCl, 4.7 KCl, 2.5 CaCl_2_, 1.2 MgSO_4_, 1.2 KH_2_PO_4_, 25.0 NaHCO_3_, and 11.1 glucose, pH 7.4) maintained at 37°C and aerated with 95%O_2_–5%CO_2_. Each ring was suspended by 2 stirrups (0.1mm diameter) to pass through the lumen. One stirrup was anchored to the bottom of the organ chamber; the other was connected to a force transducer for the measurement of isometric force. The tension of the pulmonary artery rings were monitored by a force transducer and recorded on a polygraph (Model CSF-1H; BENGBU Instruments, China). The solution in the chamber was changed every 15 minutes. Rings were allowed to equilibrate for 60 minutes at less than 1 g of resting tension. For the relaxation studies, the rings were precontracted with phenylephrine (Phe) at a concentration of 10^-5^mol/L. When the Phe-induced contraction reached a plateau level, acetylcholine (AChe, 10^-9^-10^-5^mol/L) or sodium nitroprusside (SNP, 10^-9^-10^-5^mol/L) was added in a cumulative concentration to evaluate the integrity of the endothelium. These molecules could induce substantial endothelium-dependent or endothelium-independent vasodilatation. Changes in tension induced by AChe or SNP were expressed as the percentage of the initial contraction induced by Phe. Vasodilatation percentage was calculated following the below formula:

(1) $$\text{Vasodilatation percentage}=\frac{\text{Maximal contraction tension}-\text{Vasodilatation tension}}{\text{Maximal contraction tension}-\text{Resting tension}}\times \text{100\%}$$

### Enzyme-linked immunosorbent assay (ELISA)

TNF-α concentrations from supernatants derived from lung tissue homogenates were measured using a rat TNF-α ELISA kit specific for rat cytokines according to the manufacturer’s instructions (TNF-α was from Biosource Europe SA, Belgium). Values were expressed as pg/mL.

#### Statistical analysis

Comparisons of the dose-response curves to acetylcholine, sodium nitroprusside between the two groups were made by repeated measures analysis of variance, and the *P* value for the interaction repeated measures treatment (high or low VT) is reported. Analysis was performed using commercially available software (SPSS 16.0, SPSS Inc., Chicago, IL). Data was analysed by two-way ANOVA for multiple comparisons followed by Bonferroni post-hoc test. Data for biochemical parameters were analysed by one-way ANOVA followed by Tukey's post-hoc test. Comparison between two values were made by Student's *t* test for normal distribution or Mann-Whitney sign ranked test for not-normally distributed. All values were expressed as mean ± SD. A value of *P* < 0.05 was considered to be statistically significant.

## Results

### Systemic hemodynamics

Mean artery pressure (MAP) was comparable at baseline among groups (*P* > 0.05) (Table 1). There was no significant difference in the monitored physiological variables (MAP) among the five animal groups at baseline conditions or at 0, 1, 2, 3, 4 and 5 hours after injection LPS to the groups. All values were within normal ranges for the rats. None of the MAP changed significantly at the specific studied time points among groups (*P* > 0.05) (Table 1).

**Table 1:** Effect of tidal volume on MAP of ALI rats (mmHg)

**GROUP**	**0h**	**1h**	**2h**	**3h**	**4h**	**5h**	**F**	***P***
**CON**	114.8 ± 11.0	114.8 ± 6.6	115.1 ± 3.1	117 ± 5.5	114 ± 7.8	111.6 ± 6.6	0.37	0.86
**ALI**	115 ± 5.88	117.3 ± 4.6	104.4 ± 15.1	96.6 ± 17.4	105.4 ± 13.4	101.4 ± 15.4	2.28	0.07
**LV**	113.9 ± 11.4	116.9 ± 12.7	108.8 ± 14.9	116.8 ± 12.8	115 ± 12.3	113.5 ± 16.9	0.41	0.84
**VLV**	103.8 ± 12.2	106.2 ± 13.9	105.3 ± 13.0	110.3 ± 7.3	105.8 ± 9.6	103.9 ± 13.2	0.36	0.87
**MV**	109 ± 7.9	111.2 ± 10.1	107.4 ± 8.3	110.4 ± 7.4	106.5 ± 10.4	106.1 ± 7.9	0.33	0.89
**F**	2.01	1.14	0.6	2.3	1.02	0.79		
***P***	0.1	0.36	0.7	0.07	0.42	0.56		

Hemodynamic changes. There was no difference between group and within group. CON = Control Group, ALI = Acute lung injury Group, LV = Low tidal volume Group, VLV = Very low tidal volume Group, MV = Large tidal volume Group.

### Oxygenation

PaO_2_/FiO_2_ was measured in all the groups. Compared with CON group, PaO_2_/FiO_2_ decreased 46% at the 3rd hour (361.9 ± 84.9 mmHg vs. 195.5 ± 60.6 mmHg, *P* < 0.05) in VLV group, 43% and 37.6% at the 3rd hours(385.2 ± 40.7mmHg vs. 220.3 ± 23.3 mmHg, *P* < 0.05) and the 5th hour (385.2 ± 40.7mmHg vs. 240.3 ± 25.4mmHg, *P* < 0.05) in MV group (Table 2).

**Table 2:** **Effect of tidal volume on PaO**_**2**_**/FiO2 of ALI rats (mmHg)**

**GROUP**	**0h**	**3h**	**5h**	**F**	***P***
**CON**	370.0 ± 87.0	361.9 ± 84.9	385.2 ± 40.7	0.15	0.86
**ALI**	436.5 ± 127.1	289.2 ± 121.6	272.1 ± 145.9	2.82	0.09
**LV**	321.1 ± 127.2	282.4 ± 76.0	285.0 ± 71.7	0.31	0.74
**VLV**	280.4 ± 140.8	195.5 ± 60.6*	166.4 ± 109.5*	1.78	0.2
**MV**	280.0 ± 75.0	220.3 ± 23.3	240.3 ± 25.4*	2.44	0.12
**F**	2.03	3.98	4.6		
***P***	0.12	0.012	0.006		

PaO_2_/FiO2 changes. *P < 0.05 vs. CON group. CON = Control Group, ALI = Acute lung injury Group, LV = Low tidal volume Group, VLV = Very low tidal volume Group, MV = Large tidal volume Group.

### Carbon dioxide partial pressure

The values of PaCO_2_ were comparable at baseline among groups (*P* > 0.05) (Table 2). Except for MV group which was significantly lower at the 5th hour (40.4 ± 10.7 mmHg vs. 25.2 ± 6.6 mmHg, *P* < 0.05), there was no significant difference in the monitored PaCO_2_ among the other four animal groups at 0, 3rd and 5th hours after injecting LPS within groups (Table 3).

**Table 3:** **Effect of tidal volume on PaCO**_**2**_**of ALI rats (mmHg)**

**GROUP**	**0h**	**3h**	**5h**	**F**	***P***
**CON**	49.4 ± 7.1	49.2 ± 8.3	51.3 ± 9.7	0.11	0.9
**ALI**	53.1 ± 15.9	47.9 ± 12.2	56.5 ± 26.8	0.30	0.74
**LV**	46.4 ± 18.9	42.1 ± 17.1	41.6 ± 16.4	0.14	0.87
**VLV**	49.8 ± 17.8	49.3 ± 23.0	65.9 ± 39.8	0.66	0.53
**MV**	40.4 ± 10.7	33.5 ± 7.4	25.2 ± 6.6*	4.85	0.02
**F**	0.54	0.85	1.353		
***P***	0.75	0.53	0.27		

PaCO_2_ changes. *P < 0.05 vs. 0 hour in MV group. CON = Control Group, ALI = Acute lung injury Group, LV = Low tidal volume Group, VLV = Very low tidal volume Group, MV = Large tidal volume Group.

### Pathological changes and lung injury scores

**Figure 1:**
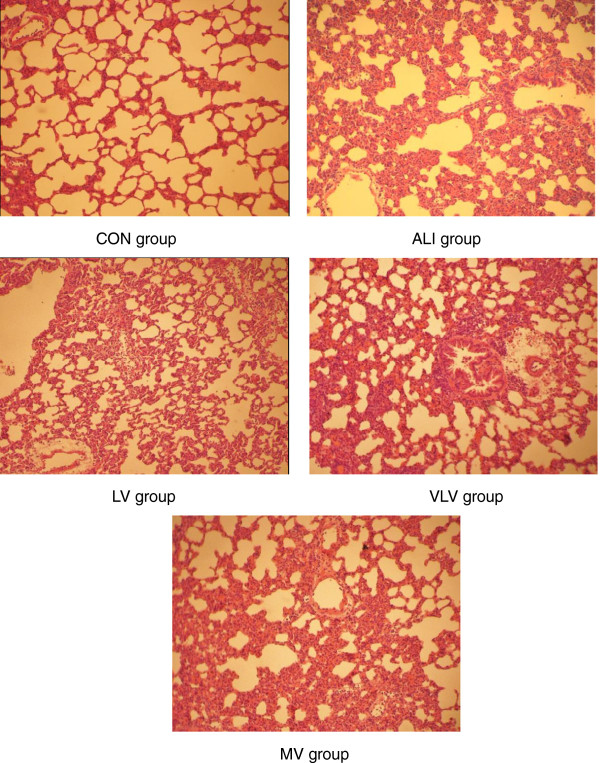
**Lung Pathological Changes.** Hematoxylin and eosin stain of representative lung tissue from rats subjected to CON=Control Group, ALI=Acute lung injury Group, LV=Low tidal volume Group, VLV=Very low tidal volume Group, MV=Large tidal volume Group. (magnification, ×200).

**Figure 2:**
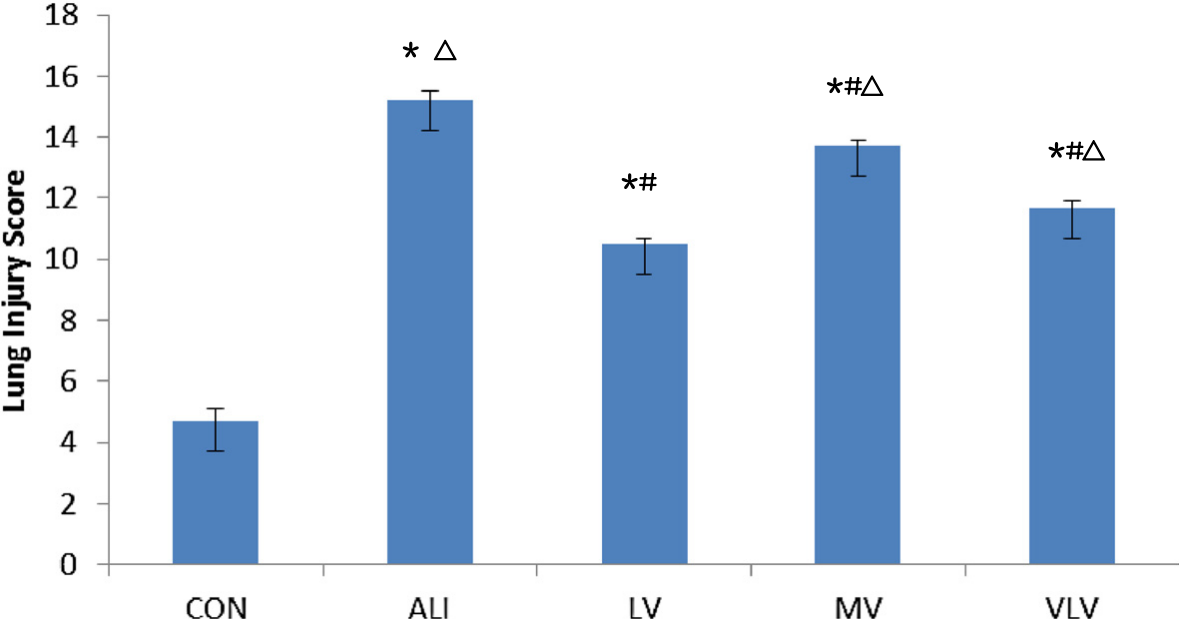
**lung Injury Scores.** *P<0.05 vs CON; ^#^*P*<0.05 vs ALI; ^△^*P*<0.05 vs LV. Six Rats/Group, Data are Mean± SD. CON=Control Group, ALI=Acute lung injury Group, LV=Low tidal volume Group, VLV=Very low tidal volume Group, MV=Large tidal volume Group.

Lung histopathology changes are shown in Figure 1. In ALI group, LPS caused lung injury, edema, congestion, thickening of the alveolar-capillary membrane, and neutrophil infiltration, while those phenomena could not be observed in the control group. Compare to ALI group, LV group showed less lung injury with less alveolar septal thickening, neutrophil infiltration and alveolar congestion (Figure 1). LV group had more protective effects than the MV and VLV groups (Figure 1). The lung injury scores were significantly higher in MV group compared to LV group (13.7 ± 0.21 vs. 10.5 ± 0.30, *P* < 0.05) and VLV group (13.7 ± 0.21 vs. 11.7 ± 0.28, *P* < 0.05) (Figure 2).

### Endothelium-dependent relaxation of isolated pulmonary artery rings

**Figure 3:**
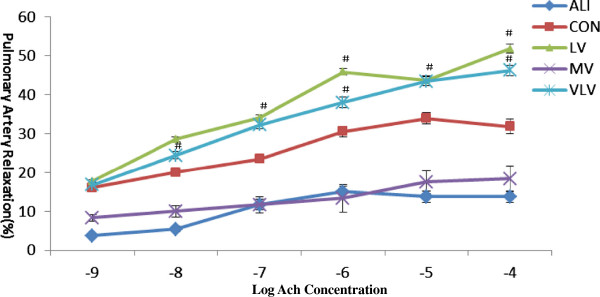
**Endothelium-dependent relaxation to Acetylcholine in isolated rat pulmonary artery rings.** Ach= Acetylcholine. *P<0.05 vs CON; ^#^*P*<0.05 vs ALI; ^△^*P*<0.05 vs LV; Six Rats/Group. CON=Control Group, ALI=Acute lung injury Group, LV=Low tidal volume Group, VLV=Very low tidal volume Group, MV=Large tidal volume Group.

**Figure 4:**
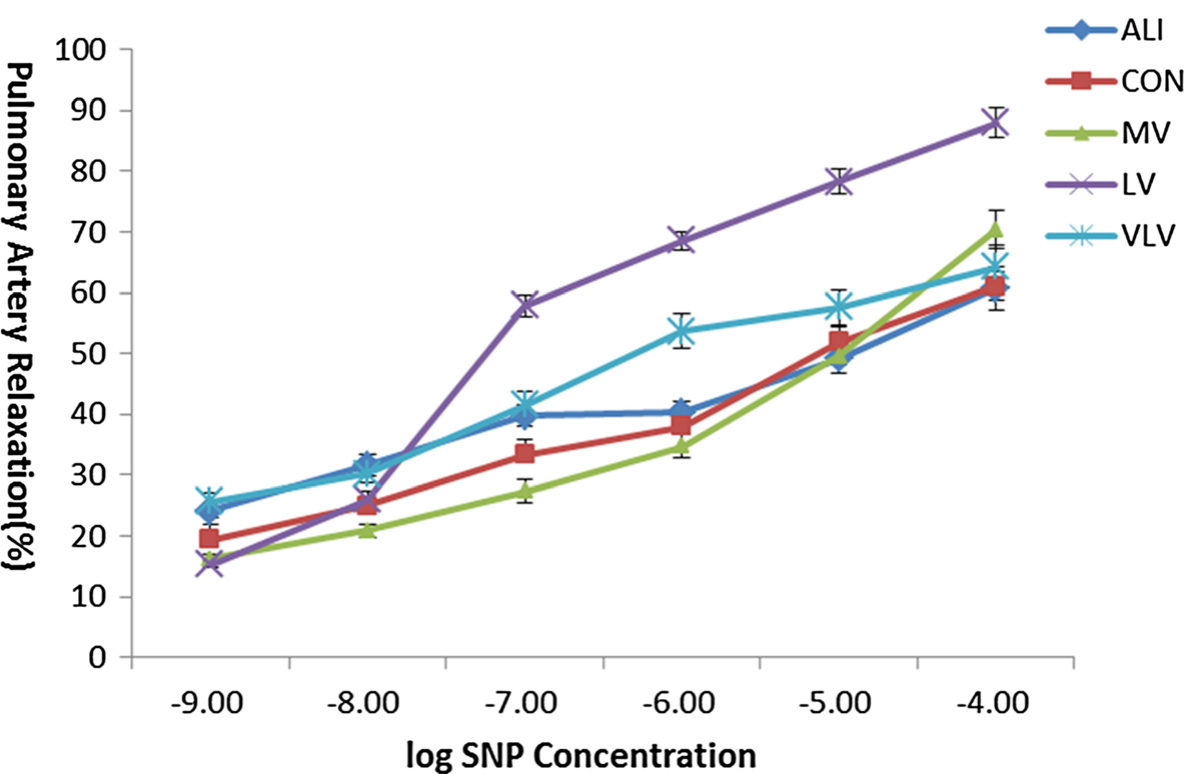
**Endothelium-independent relaxation response to sodium nitroprusside in isolated rat pulmonary artery rings.** SNP= sodium nitroprusside. No difference of endothelium-independent relaxation to sodium nitroprusside among the groups. Six Rats/Group. CON=Control Group, ALI=Acute lung injury Group, LV=Low tidal volume Group, VLV=Very low tidal volume Group, MV=Large tidal volume Group.

Concerning endothelium-dependent vasomotor function, Ache (10^-9^-10^-5^mol/L) resulted in a concentration-dependent relaxation from phenylephrine-induced precontraction in pulmonary artery rings among all groups (Figure 3). But the percentages of relaxation of artery rings were different when stimulated by Ache at different concentrations. Ache response was significantly reduced after LPS treatment. Compared with the control group, the percentage of relaxation of pulmonary artery rings was significantly decreased in LPS treated rats, and the maximal relaxation was decreased by 18% in 10^-4^mol/L of Ache (*P* < 0.05) (Figure 3). But, LPS-induced decrease of percentage of relaxation was improved in the pulmonary artery rings harvested from animals with VLV group and LV group (Figure 3). Compared with MV group, LV group improved relaxation to Ache (10^-7^-10^-5^mol/L) more significantly, which was about 22%-33% (*P* < 0.05) (Figure 3). However, the maximum of endothelium-independent relaxation to SNP (10^-9^-10^-5^mol/L) was not influenced by any group (*P* > 0.05) (Figure 4).

### Level of ET-1 in lung tissue

**Figure 5:**
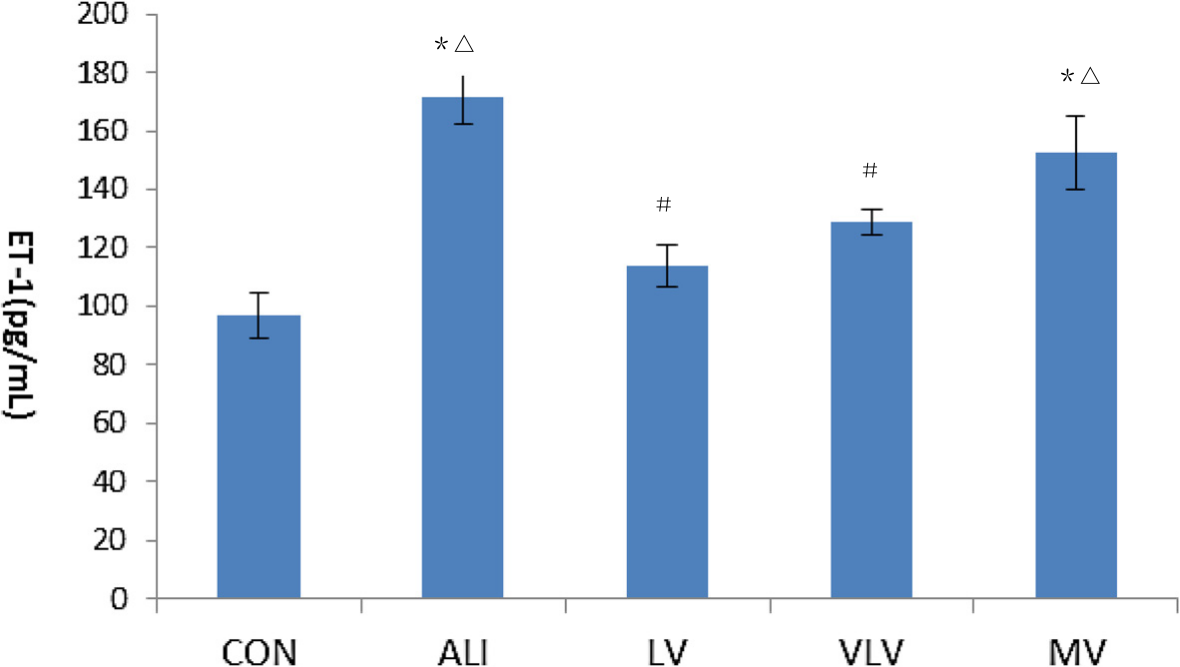
**Level of ET-1 in lung tissue of rats (pg/mL).** fET-1: endothelin-1. *P<0.05 vs CON; ^#^*P*<0.05 vs ALI; ^△^*P*<0.05 vs LV. Six Rats/Group, Data are Mean± SD. Six Rats/Group. CON=Control Group, ALI=Acute lung injury Group, LV=Low tidal volume Group, VLV=Very low tidal volume Group, MV=Large tidal volume Group.

After injection of LPS, the levels of ET-1 in lung tissues were significantly increased in ALI rats (Figure 5). While the levels of ET-1 in lung tissues were significantly decreased both in LV group (171.8 ± 9.22pg/mL vs. 113.79 ± 7.33 pg/mL, *P* < 0.05) and VLV group (171.8 ± 9.22 pg/mL vs. 128.54 ± 4.37 pg/mL, *P* < 0.05). The concentration of ET-1 was also lower in LV group compared to the value in MV group (113.79 ± 7.33 pg/mL vs. 152.52 ± 12.75 pg/mL, *P* < 0.05).

### Expression of eNOS protein in lung tissues

**Figure 6:**
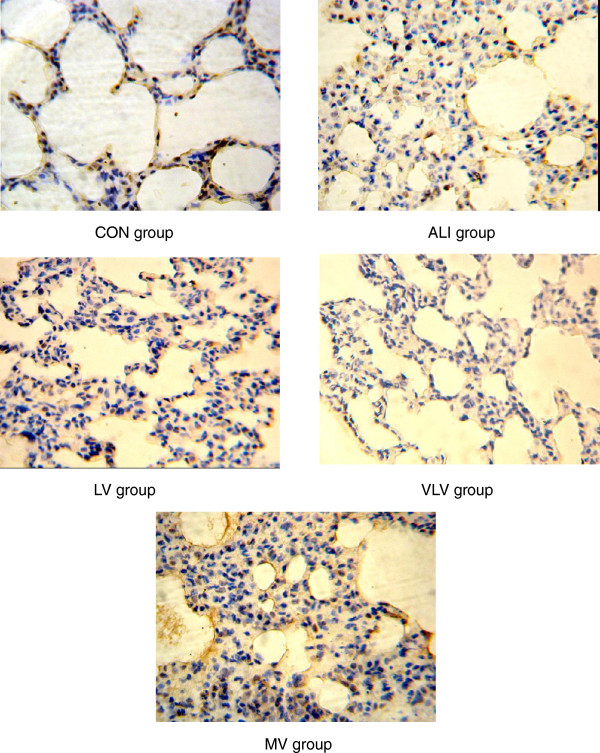
**eNOS protein in pulmonary tissues.** The pulmonary tissues were analyzed for expression of eNOS protein by immunohistochemistry(IHC). Dark brown staining of indicates eNOS-positive cells. IHC stain of representative pulmonary artery endothelial cells from rats subjected to CON=Control Group, ALI=Acute lung injury Group, LV=Low tidal volume Group, VLV=Very low tidal volume Group, MV=Large tidal volume Group (magnification, ×400).

**Figure 7:**
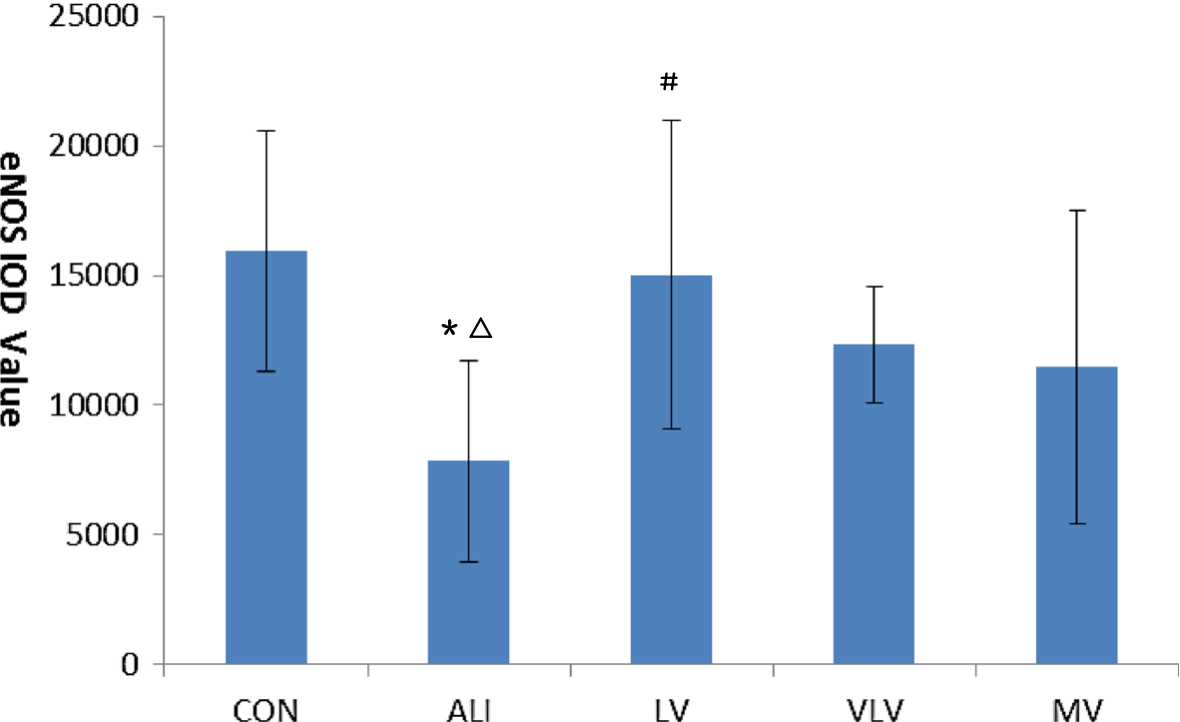
**IOD values of eNOS protein in pulmonary tissue of rats with ALI.** IOD:integraloptical density. *P<0.05 vs CON; ^#^*P*<0.05 vs ALI; ^△^*P*<0.05 vs LV. Six Rats/Group, Data are Mean± SD. Six Rats/Group. CON=Control Group, ALI=Acute lung injury Group, LV=Low tidal volume Group, VLV=Very low tidal volume Group, MV=Large tidal volume Group.

Expressions of eNOS protein in lung tissues are shown in Figure 6. Immunohistochemistry revealed that eNOS protein was mainly localized in endothelial cells. Abundant expression of eNOS protein in lung tissues in the control group was observed. Expression of eNOS protein was lower in the ALI group than that of the control group (7831.03 ± 3892.51 vs. 15919.86 ± 4637.23, *P* < 0.05) (Figure 7). Compared with the ALI group, LV group significantly increased the expression of eNOS protein in the pulmonary artery endothelium (7831.03 ± 3892.51 vs. 15032.05 ± 5925.07, *P* < 0.05) (Figure 7).

### Level of TNF-α in lung tissues

**Figure 8:**
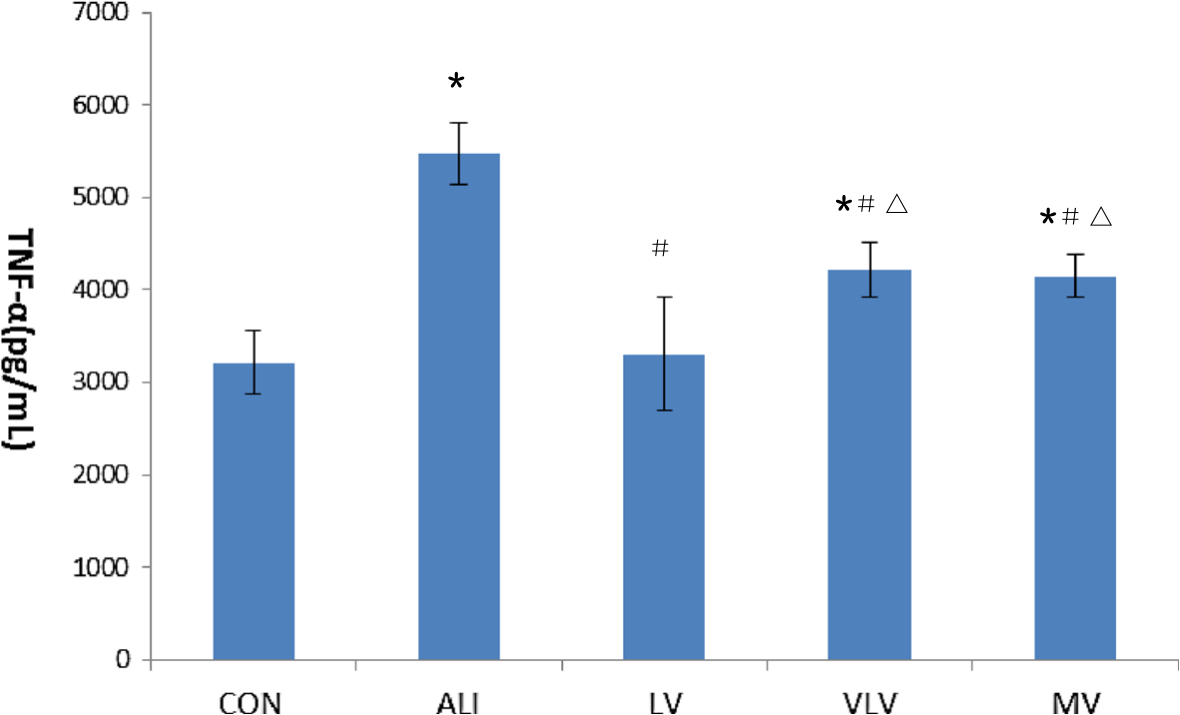
**TNF-α in pulmonary tissue (pg/mL).** TNF-α: tumor necrosis factor. *P<0.05 vs CON; ^#^*P*<0.05 vs ALI; ^△^*P*<0.05 vs LV. Six Rats/Group, Data are Mean± SD. Six Rats/Group. CON=Control Group, ALI=Acute lung injury Group, LV=Low tidal volume Group, VLV=Very low tidal volume Group, MV=Large tidal volume Group.

ALI rats were associated with a significant increase of TNF-α level in the lung tissues after LPS injection. In contrast, LPS-induced increase of TNF-α level in the lung tissues was significantly blunted in LV group compared with that in the MV group (3305.09 ± 608.21 pg/mL vs. 4144.07 ± 235.4 pg/mL, *P* < 0.05) (Figure 8).

## Discussions

Although previous studies have demonstrated that high tidal volume ventilation of injured lungs increased pulmonary hypertension and may result in the increased mortality, the effects of low tidal volume ventilation on lung endothelial injury and lung vascular vasomotor have not been clarified. The objective of this study was to determine if tidal volume reduction could improve pulmonary vasomotor tone by decreasing lung endothelial cell injury in the LPS-induced lung injured rat. By detecting and analyzing the biochemical, functional, and histological markers of injury, we found that ventilation with 6 mL/kg and 3 mL/kg tidal volume could protect the lung vascular diastolic function from ALI.

Sepsis is an independent risk factor of ALI/ARDS. When the septic focus is extrapulmonary, it constitutes 33% of indirect ALI/ARDS [13,14]. We found that the sepsis induced by LPS could impaire endothelial barrier integrity and induce the imbalance of vasoactive compounds, such as increased ET-1 level(ALI group vs. CON group, 171.8 ± 9.22 vs. 96.58 ± 7.81, *P* < 0.05), decreased eNOS expression(ALI group vs. CON group, 7831.03 ± 3892.51 vs. 15919.86 ± 4637.234, *P* < 0.05), and decreased pulmonary artery vasodilatation.

The pulmonary endothelium is a continuous monolayer of squamous cells that internally lines the blood vessels. It is a major metabolic organ promoting adequate pulmonary and systemic vascular homeostasis [15]. The mechanisms of regulating pulmonary vasomotor are: (1) endothelium releases vasoactive mediators, which regulate the physical and biochemical properties of the pulmonary vessels and affect vascular contractility [16]. (2) Endothelial dysfunction contributes to the thrombotic process, and induces vascular luminal obliteration [17]. (3) The loss of endothelial barrier integrity may provide a surreptitious avenue for platelets to contact with the subendothelial structures, resulting in release of vasomediators [18]. In our study, we found that LPS not only changed the releases of endothelium vasoactive mediators, such as decreased eNOS expressions and increased ET-1 concentration, but injured endothelial barrier integrity which induced lung edema.

Mechanical ventilation could improve hypoxemia, maintain lung volume and promote alveolar opening. Low tidal volume is advocated for ALI patients. Many studies proved that high tidal volume could not only induce high permeability pulmonary edema in normal and injured lung in different signal pathways [19,20], but decrease vascular relaxations to acetylcholine [5,21], sometimes it was considered to be the “second hit” to ALI patients. According to our results, Ventilation with the tidal volume of 6 mL/kg resulted in significantly less lung injury and vasodilatation compared with 12 mL/kg and sepsis induced ALI groups. This may be the result of a reduction in the severity of lung endothelial injury. We found that level of TNF-α and ET-1 was higher in 12mL/kg and sepsis induced ALI groups, and These mean that 6mL/kg tidal volume could decrease cyclic stretch(alveolar tidal opening-closing and hyperinflation), inflammatory response and protect endothelium-dependent relaxation from LPS-induced lung injury. The reasons could be inferred as follows: (1) Cyclic stretch could induce system inflammation, injury endothelial cells [21-23], and impair endothelium-dependent vasodilatation. (2) Cyclic stretch could cause an increase in production of various vasoactive substances in endothelial cells, and the ET-1 mRNA levels increased in response to fluid cyclic stretch [24]. Endothelin (ET)-1 is a potent vasoconstrictor peptide mainly synthesized and released from injured vascular endothelium [25]. It is also abundantly synthesized in the lung to produce pulmonary vasoconstriction. Clinical studies found that in patients with sepsis and ALI/ARDS, ET-1 was produced mainly in the lung and was not only associated with pulmonary vasoconstriction but also the development of permeability oedema and patients’ prognosis [26]. In our study, compared with 6 mL/kg tidal volume group, we found ET-1 concentration was high in 12mL/kg tidal volume group (113.79 ± 7.33 pg/mL vs. 152.52 ± 12.75pg/mL, *P* < 0.05), and it was one reason which impaired the pulmonary vasomotor function. (3) Inflammatory response is another reason that injure the endothelial function. Under inflammatory conditions, endothelial cell activation occurs, leading to a loss of vascular integrity, increased expression of leucocyte adhesion molecules, a change in phenotype from anti- to pro-thrombotic, cytokine production and endothelium vasoactive mediators release [27]. In our study, we found that ALI and 12mL/kg tidal volume could increase TNF-α(5467.51 ± 334.29pg/mL in ALI group, 4144.07 ± 235.4pg/mL in 12mL/kg tidal volume group), and increase inflammatory response. Although the tidal volume of 12mL/kg had revealed the harmful effects in ALI, the effect of the high tidal volume on vasodilatation was similar with the ALI group, the tidal volume higher than 12mL/kg need to be further studied, and it might be worsen in vasodilatation than the ALI group.

Very low tidal volume seems to have the same effects on pulmonary vasomotor tone, compared with low tidal volume. However, Terragni PP, et al. found that Vt lower than 6 mL/kg enhanced lung protection, but respiratory acidosis was obvious in the ALI patients with very low tidal volume, and extracorporeal carbon dioxide removal was needed to rectify respiratory acidosis [28]. Another problem of lower tidal volume is that the increase in respiratory rate used to avoid low tidal volume reduction-induced severe hypercapnia, but high respiratory rate may also induce substantial gas trapping and PEEPi in patients with ALI [29]. We found the PaCO_2_ in very low tidal volume group (65.9 ± 39.8mmHg) is higher than the other groups, although there is no statistic difference, and severe gas trapping was not found in histopathology. By analyzing the measured vasoactive substances and vasomotor function, we found very low tidal volume could protect lung from injury, but respiratory acidosis should be considered.

Sodium nitroprusside (SNP) is one of the most widely studied nitric oxide donors, and is used to be an exogenous nitric oxide donors in our study. The effects of NO release depend on its interaction with sulfhydryl-containing molecules in vivo, the mechanism is not influenced by endothelium function [30]. As Nin’s study said, lung inflammatory response stimuli seemed likely to affect arterial vascular function through impaired smooth muscle function or through decreased availability of nitric oxide [5]. But under our experiment conditions, there was no statistic differences in endothelium-independent dilation function among groups. Different results appeared might be because of the different tidal volumes used in the high tidal volume groups (12mL/kg in our study vs. 35mL/kg in Nin’s study).

Previous clinical studies have shown that low tidal volume in ALI/ARDS patients correlated with reduced mortality [7], and lung endothelial injury correlated with the severity of lung injury and dysfunction of pulmonary vasomotor tone [31]. Therefore, the reduced injury to the lung endothelium in this study may help to explain the benefits observed in clinical trials of low tidal volume ventilation. Furthermore, it is possible to use the lung protective strategy at a tidal volume lower of 6 mL/kg to provide protection to the alveolar endothelium.

## Conclusions

In conclusion, this study suggested that low tidal volume ameliorated pulmonary vasomotor tone, inhibited the inflammation factors and protected pulmonary endothelial dependent vasomotor dysfunction from “second hit” in indirect ALI rats. Additional clinical studies are required to further confirm the applications of these findings.

## Competing interests

The authors declare that they have no competing interests.

## Authors’ contributions

Chun Pan conceived of the study, participated in its design and animal experiment and drafted the manuscript. Jian-qiang Wang carried out the animal experiment, participated in the sequence alignment and helped to draft the manuscript. Wei Liu helped to draft and modified the manuscript. Ling Liu carried out the biochemical and histological experiment. Liang Jing participated in the pulmonary artery rings tension test. Yi Yang participated in the design of the study and performed the statistical analysis. Haibo Qiu participated in the design and coordinated the study. All authors read and approved the final manuscript.
